# The effect of different divalent cations on the kinetics and fidelity of *Bacillus stearothermophilus* DNA polymerase

**DOI:** 10.3934/biophy.2018.2.125

**Published:** 2018-04-25

**Authors:** Ashwani Kumar Vashishtha, William H. Konigsberg

**Affiliations:** 1 Centre for Structural and Functional Genomics, Concordia University, Montreal, Canada; 2 Yale University, 333 Cedar St., SHM C-E14, New Haven, CT, 06520, USA

**Keywords:** divalent cations, base selectivity, BST pol, kinetics

## Abstract

Although Mg^2+^ is the metal ion that functions as the cofactor for DNA polymerases (DNA pols) *in vivo*, Mn^2+^ can also serve in this capacity but it reduces base discrimination. Metal ions aside from Mg^2+^ or Mn^2+^ can act as cofactors for some DNA pols but not for others. Here we report on the ability of several divalent metal ions to substitute for Mg^2+^ or Mn^2+^ with BST DNA polymerase (BST pol), an A family DNA pol. We selected the metal ions based on whether they had previously been shown to be effective with other DNA pols. We found that Co^2+^ and Cd^2+^ were the only cations tested that could replace Mg^2+^ or Mn^2+^. When Co^2+^ was substituted for Mg^2+^, the incorporation efficiency for correct dNTPs increased 6-fold but for incorrect dNTPs there was a decrease which depended on the incoming dNTP. With Mn^2+^, base selectivity was impaired compared to Co^2+^ and Cd^2+^. In addition, Co^2+^ and Mn^2+^ helped BST pol to catalyze primer-extension past a mismatch. Finally both Co^2+^ and Mn^2+^ enhanced ground-state binding of both correct and incorrect dNTPs to BST pol: Dideoxy terminated primer-template complexes.

## 1. Introduction

DNA polymerases replicate genomic DNA with extremely high fidelity [1]. A two metal ion catalytic mechanism has been widely accepted for DNA pols [2]. All DNA polymerases known to date require two and sometimes three divalent cations (usually Mg^2+^) for the nucleotidyl transfer reaction but only two are needed to catalyze the 3′→5′ exonuclease activity associated with replicative DNA pols. Even though DNA pols utilize the physiologically relevant Mg^2+^, Mg^2+^ can substitute for Mg^2+^ in the nucleotidyl transfer reaction but, when this occurs, it tends to lower the base selectivity dramatically [3–6]. Two metal ions play an important role in assembling the catalytic groups, the metal ion present in the “A” site helps to lower the p*K*a of the terminal 3′ OH group on the primer-terminus and coordinates both the 3′-OH of the primer strand and the α-phosphate of incoming dNTP facilitating the nucleophilic attack of the 3′-OH on the α-phosphorous of incoming dNTPs [7]. The metal ion in the “B” site coordinates the α-, β-, and γ-phosphate oxygens of the incoming dNTPs, assists in neutralizing the developing negative charge in the transition state, and assists the departure of the PP_i_ product. The third metal ion likely helps to neutralize the negative charge built up in the transition state and may also help in protonating the leaving PP_i_ [8]. The effect of metal ion cofactors on the fidelity of DNA replication has been studied for various pols, including *E. coli* DNA pol I [9], AMV DNA polymerase [10], Klenow fragment of *E. coli* DNA pol I [11], T4 polymerase [12], T7 DNA polymerase [13], human DNA polymerase α, human DNA polymerase β [13], *Sulfolobus solfataricus* DNA pol IV (Dpo4) [3] and RB69pol [14]. A number of metal ions have been shown to be mutagens and carcinogens and some may act by reducing the accuracy of DNA replication [15], although there are many other possibilities that would produce the same results such as interference by metal ions in DNA repair pathways [16].

The effect of metal ions on the fidelity of DNA polymerases is still not well understood [3,9–13,17]. Irima et al. have reported that Mn^2+^ and Ca^2+^ can act as cofactors for Dpo4-catalyzed polymerization but Co^2+^, Ni^2+^, Cu^2+^, Zn^2+^, Ba^2+^, and Sr^2+^ do not [6]. Pelletier et al., 1996 have studied the primer extension using pol β and showed that when crystals of pol β complexed with DNA were soaked in the presence of Ca^2+^, Mn^2+^, Co^2+^, Ni^2+^, Cu^2+^, Zn^2+^, Cd^2+^, and Cr^3+^, only Mn^2+^, Cd^2+^, and Zn^2+^ allowed the reaction to occur in the crystals [4]. We have recently carried out pre-steady-state kinetic analysis using RB69pol and showed that, apart from Mg^2+^, Mn^2+^, Co^2+^, and to a lesser extent Ni^2+^, these metal ions can support nucleotidyl transfer as well as the exonuclease activity of RB69pol [14]. Dpo4, Pol β and RB69pol belong to the Y-, X-, and B-families respectively, and have different preferences for divalent cations, however since there are no reports on extensive studies involving the effect of various divalent cations on the kinetics and fidelity of A-family pols we decided to determine the metal-ion cofactor preference for BST pol a high fidelity A-family DNA polymerase [18,19].

Here we report on the different divalent cations that support nucleotidyl transfer by BSTpol and we show how these divalent cations impact base selectivity by measuring the pre-steady-state kinetic parameters for primer extension with correct/incorrect incoming dNTPs. We have also determined the ground-state binding affinity of dTTP and dCTP opposite 2AP, as the templating base, in the presence of various divalent cations by observing the quenching of 2AP fluorescence as a function of [dNTP]. We have also looked at the ability of different divalent metal ions to support extension past a mismatched primer/template terminus. We have attempted to explain why BST pol can utilize only selected divalent cations as cofactors based on the properties of these metal ions and we have compared our results with other DNA pols.

## 2. Materials and methods

### Chemicals

All chemicals were of the highest grade available and were used as purchased. dNTPs were from Roche (Burgess Hill, UK), [γ-32P]ATP was from MP Biomedicals (Irvine, CA), and T4 polynucleotide kinase was from New England Biolabs (Ipswich, MA). The metal ion salts (BaCl_2,_ CaCl_2_, CdSO_4_, CoCl_2_, CuSO_4_, CrKSO_4_, MgCl_2_, MnCl_2_, NiCl_2_, SrCl_2_, FeCl_2_, and Zn(CH_3_COO)_2_) were from Fluka and were >99% pure.

### DNA substrates and enzymes

**Figure 1:**
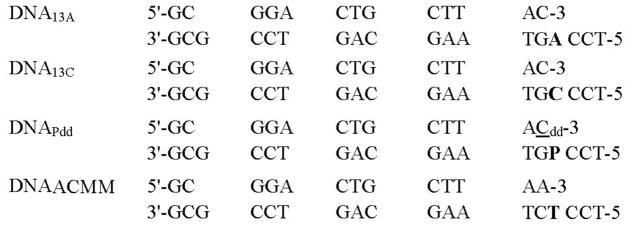
Primer/template sequences used for the pre-steady-state kinetic assays and equilibrium fluorescence titrations. The templating base is in bold. P represents 2-aminopurine as the templating base; C represents the dideoxy-terminated cytosine.

All oligonucleotides used in this study were synthesized at the Keck facilities (Yale University). All primer strands were gel purified using PAGE (19:1% (w/v) acrylamide: Disacrylamide gels containing 8 M urea). The primer was labeled with [γ-32P]-ATP using T4 polynucleotide kinase and was annealed to unlabeled templates (in a 1:1.1 ratio) by heating to 95 °C, followed by slowly cooling to 25 °C [20]. For equilibrium fluorescence titration studies using 2AP as the templating base, the primer was annealed to the template strand containing 2AP at the templating position in a 1.1:1 ratio. The P/T sequences used in all assays are shown in Figure 1. In order to simplify data interpretation, a common primer sequence was annealed to different templates where the templating base was varied. BST pol was purified as reported earlier [21].

### Steady-state kinetic assays for metal ion dependence of BST pol

Steady-state kinetic analysis was used to investigate the ability of various metal ions to support the polymerase activity of BST pol. A typical assay mixture contained 200 nM 13/18-mer (DNA_13A_), 40 nM BST pol enzyme and 10 mM MgCl_2_ in 66 mM Tris-HCl (pH 7.3). The mixture was preincubated at 23 °C for 10 min and the reaction initiated by adding 500 μM dTTP. 5 μL aliquots were withdrawn at various times (10, 20, 40, 60, and 120 s) and quenched with 0.5 M EDTA. The assay was repeated by replacing MgCl_2_ with BaCl_2,_ CdSO_4_, CuSO_4_, CrKSO_4_, FeCl_2_, MnCl_2_, NiCl_2_, SrCl_2_, and Zn(CH_3_COO) [2].

### Single-turnover incorporation assays for correct/incorrect incoming dNTPs catalyzed by BST pol

Rapid-chemical quench assays were performed at 23 °C with RQF3 rapid chemical quench instrument (Kintek Corporation). 1 μM BST pol was incubated with 80 nM 13/18-mer P/T (DNA_13A_) in assay buffer containing 66 mM Tris-HCl (pH 7.3), EDTA (0.1 mM), and mixed with dTTP (10–500 μM) containing MgCl_2_, MnCl_2_, CoCl_2_, or CdSO_4_, (10 mM) in assay buffer (final concentrations after mixing). The reactions were quenched with 0.5 M EDTA (pH 8.0) at various times ranging from 4–700 ms. For an incorrect nucleotide incorporation, the dNTP concentrations were varied from 50 μM to 3 mM and reactions were quenched at times ranging from 1–280 min. DNA Products were separated by PAGE (19:1% (w/v) acrylamide: Disacrylamide gels containing 8 M urea), visualized using a STORM imager (Molecular Imaging) and quantified using ImageQuant (GE Healthcare) and GraphPad Prizm.

### Single-turnover incorporation assays for insertion and extension beyond a mismatched P/T

Single turnover experiments were carried out as described above with the exception that DNA_13A_ was replaced with DNA_ACMM_ as shown in Figure 1. 1 μM BST pol was incubated with 80 nM DNA_ACMM_ in assay buffer containing EDTA (0.1 mM) and mixed with dATP (0.05–3 mM) containing 10 mM each Mg^2+^, Co^2+^, Mn^2+^ or Cd^2+^, in the assay buffer. The reactions were quenched with 0.5 M EDTA (pH 8.0) at various times ranging from 3 s to 80 min. Five different [dATP] were employed for each *k*_pol_ and *K*_d,app_ determination.

### Determination of K_dg_ for incoming dTTP/dCTP using equilibrium fluorescence titrations

The emission spectra of dideoxy-primer/templates (ddP/T) containing 2AP at the templating position were recorded at 23 °C with a Photon Technology International Alphascan scanning spectrofluorometer. The assay contained 200 nM P/T (DNA_Pdd_) (Figure 1), 1 μM BST pol, and either 10 mM MgCl_2_, 10 mM CoCl_2_, 10 mM MnCl_2_, or 10 mM CdSO_4_ with varying [dTTP] and [dCTP]. Samples were excited at 313 nm and fluorescence emission spectra were collected from 335 to 450 nm. All intensities for all samples were normalized by subtracting the intensity of the blank sample and protein which contained 66 mM Tris-HCl (pH 7.3) and the metal ion. Peak emission intensities at 365 nm were plotted as a function of [dTTP] (or [dCTP]) and fit to a hyperbolic equation to obtain the ground-state equilibrium dissociation constant (*K*_dg_) for incoming dTTP (or dCTP).

### Data analysis

The amount of product formed at each [dNTP] at different times was fit by non-linear regression to Eq 1 and the observed rates of product formation at different [dNTP] were determined.

(1) $$[\text{Product}]=A\left[1-e^{-k_{\mathrm{obs}}t}\right]$$

Where *k_obs_* is the observed rate constant at a particular [dNTP], and *A* is the observed amplitude of product formation and *T* is time. To determine the pre-steady-state kinetic parameters *k*_pol_ and *K*_d,app_, *k*_obs_ was plotted against the [dNTP] and data were fitted to Eq 2.

(2) $$k_{\mathrm{obs}}=\frac{k_{\text{pol}}[\mathrm{dNTP}]}{K_{d,\mathrm{app}}[\mathrm{dNTP}]}$$

Where *k*_obs_ is the observed rate at a given [dNTP]. In cases where saturation was not achieved with the incoming dNTP, data were fit to Eq 3.

(3) $$k_{\mathrm{obs}}=\frac{k_{on}[\mathrm{dNTP}]}{1+[\mathrm{dNTP}]/K_{d,\mathrm{app}}}$$

Where *k*_on_ is defined as the ratio of *k*_pol_ to the *K*_d,app_ value for the incoming dNTP. Equilibrium fluorescence titration data were fit to the hyperbolic equation.

(4) $$f=\frac{f_{\max}\;[\mathrm{dNTP}]}{K_{d,g}+[\mathrm{dNTP}]}$$

Where *f* is the observed fluorescence intensity and *f*_max_ is the maximum decrease in fluorescence intensity at saturating [dTTP] and *K*_d,g_ is the ground-state equilibrium dissociation constant for the incoming dNTP.

## 3. Results

The effect of divalent metal ions on the replication fidelity of various pols has been studied previously but the rates of incorporation of correct and incorrect nucleotides have not been determined under single-turnover conditions in the presence of different metal ion cofactors except for Mn^2+^ [2,22] and Co^2+^ [17]. Co^2+^, Mn^2+^, and Ni^2+^ have been characterized as “mutagens”, since they reduce the fidelity of DNA synthesis [3,4,9–13]. Attempts have been made in the past to find out why these metal ions foster the mutagenic behaviors of DNA pols but most of these efforts have focused on Mn^2+^ [11,12,22]. Metal ions can affect the behavior of high fidelity polymerases by altering the various checkpoints along the reaction pathway. For example: 1) metal ions can affect the ground-state binding affinity of the correct and incorrect dNTPs to pol/P/T binary complexes; 2) they can promote misincorporation during primer extension; 3) intrinsic exonuclease activity can be diminished resulting in the failure to remove incorrectly incorporated dNMPs; 4) the efficiency of extension beyond the mismatch could be affected upon encountering a mismatch at the P/T terminus, resulting in mutations being embedded in the DNA; 5) the metal ion could also affect enzymes in the DNA repair pathways [16]. Here, we used BST pol, a high fidelity A family DNA replicative polymerase, to systematically study the effects of various divalent metal ions on the fidelity checkpoints during DNA replication.

### Mg^2+^, Mn^2+^, Co^2+^, and Cd^2+^ can support primer extension catalyzed by BST pol

Steady-state kinetic assays were carried out with a number of divalent cations including Mg^2+^, Mn^2+^, Co^2+^, Ni^2+^, Fe^2+^, Ca^2+^, Zn^2+^, Cd^2+^, Sr^2+^, Ba^2+^, Cu^2+^, and Cr^3+^ to determine which ones were able to activate BST polymerase. Our choice of these metal ions was based on the fact that those stated above were shown to activate other pols [4,6]. We found that apart from Mg^2+^ and Mn^2+^ only Co^2+^ and Cd^2+^ supported the pol activity of BST pol. Table 1 summarizes the effect of the different metal ions on the activity of BST pol. We used 10 mM each of Mg^2+^, Mn^2+^, Co^2+^, and Cd^2+^ for pre-steady-state single-turnover experiments to investigate their effects on misincorporation and their ability to allow BST pol to extend a primer-terminus beyond a mismatch.

### Single-turnover kinetics for correct nucleotide incorporation by BST pol

**Figure 2:**
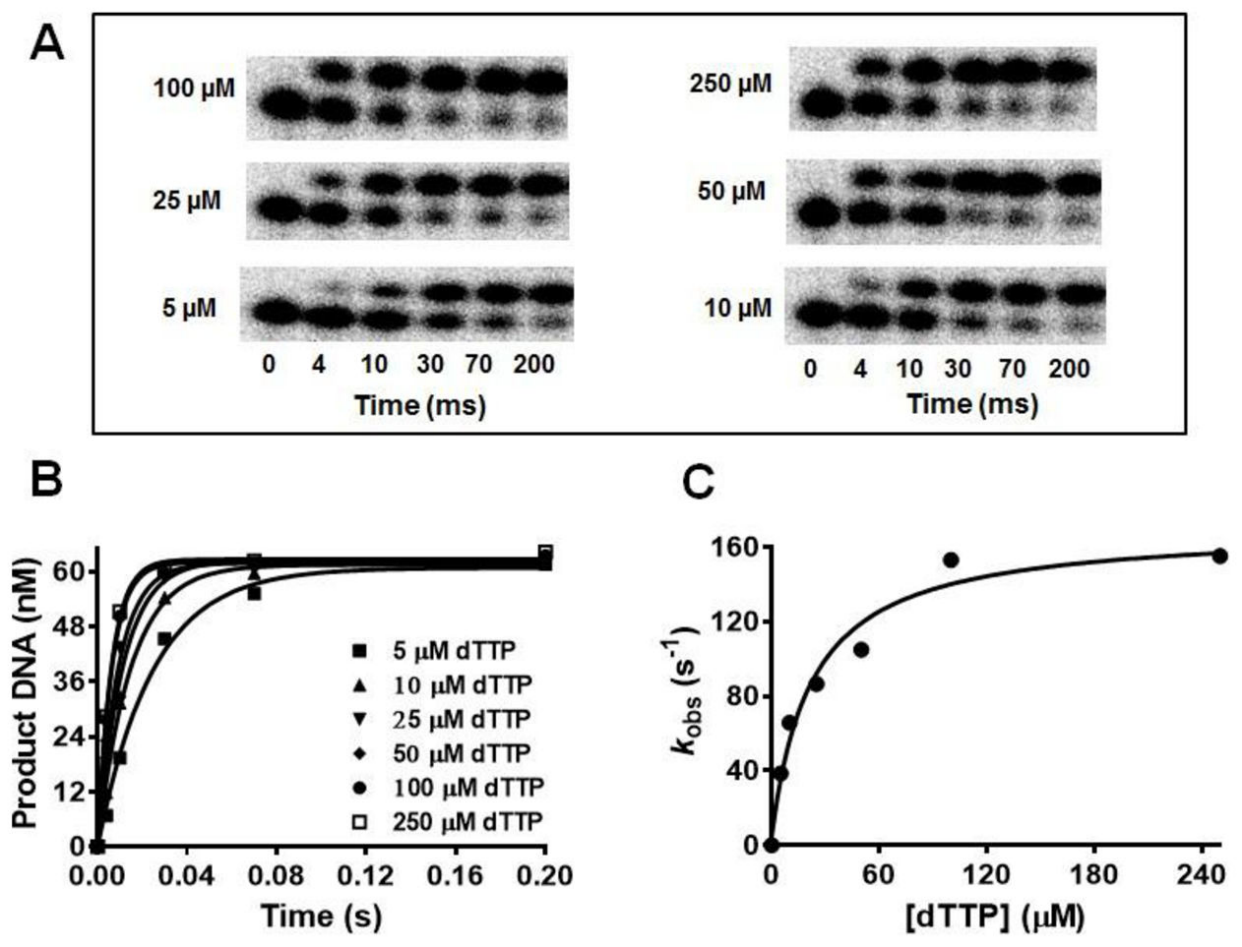
Concentration dependence of the rate of dTTP incorporation opposite dA as the templating base with Mn^2+^. BST pol (1 μM) was pre-incubated with DNA_13A_ (80 nM) in reaction buffer and was mixed with increasing concentrations of dTTP [5, 10, 25, 50, 100, and 250 μM] containing 10 mM Mn^2+^. Reactions were quenched with 0.5 M EDTA (pH 8.0) at various times ranging from 4–200 ms. All data were obtained at 23 °C. (A) Gel images showing dTMP incorporation opposite dA at various [dTTP]; (B) plots of the amount of extended DNA product obtained as a function of time at various [dTTP]. Points are experimental, while curves are based on a fit of the data to Eq 1; (C) the single exponential rates obtained were plotted as a function of [dTTP] and fitted to Eq 2 to obtain a *k*_pol_ of 166 ± 14 s^−1^ and a *K*_d,app_ of 22 ± 7 μM.

**Figure 3:**
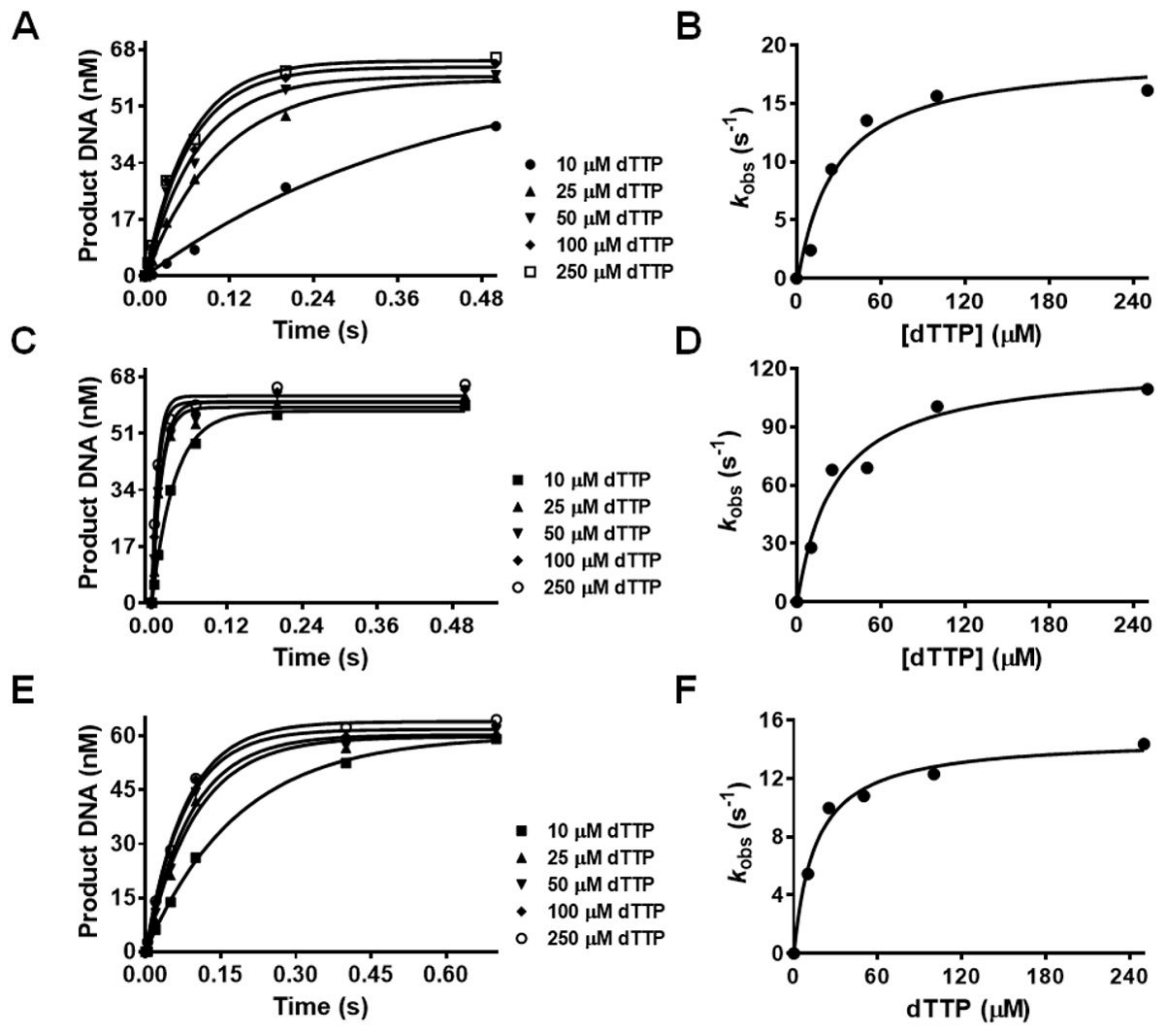
Concentration dependence of the rate of dTTP incorporation opposite dA as the templating base with Mg^2+^, Co^2+^, and Cd^2+^. BST pol (1 μM) was preincubated with DNA_13A_ (80 nM) in reaction buffer and was mixed with increasing concentrations of dTTP [10, 25, 50, 100, and 250 μM] containing 10 mM Mg^2+^, 10 mM Co^2+^, or 10 mM Cd^2+^, respectively. Reactions were quenched with 0.5 M EDTA (pH 8.0) at various times ranging from 4–500 ms (Mg^2+^), 4–500 ms (Co^2+^), and 4–700 ms (Cd^2+^). All data were obtained at 23 °C. (A), (C), (E) plots of the amount of extended DNA product obtained as a function of time at various [dTTP] with 10 mM Mg^2+^, Co^2+^ or Cd^2+^ respectively. Points are experimental, while curves are based on a fit of the data to Eq 1. (B), (D), (F) the single exponential rates obtained were plotted as a function of [dTTP] and fitted to Eq 2 to obtain a *k*_pol_ of 20 ± 2 s^−1^ and a *K*_d,app_ of 26 ± 11 μM with 10 mM Mg^2+^, a *k*_pol_ of 123 ± 12 s^−1^ and a *K*_d,app_ of 28 ± 9 μM with Co^2+^, or a *k*_pol_ of 15 ± 1 s^−1^ and a *K*_d,app_ of 15 ± 3 μM with Cd^2+^ respectively.

Even though Mg^2+^ activates BST pol *in vivo* and is the physiologically relevant cation for almost all DNA pols, DNA polymerases from human [23], viral [24] and bacterial [9] sources can also utilize Mn^2+^, Co^2+^, Ni^2+^, and Cd^2+^ *in vitro* [4]. Dpo4 [6] is the only pol which can be activated by Ca^2+^ but this is an exception since Ca^2+^ is inactive with every other DNA pol that has been tested. We used single-turnover conditions to determine the apparent dissociation constant for dTTP (*K*_d,app_) in the presence of Mg^2+^, Mn^2+^, Co^2+^, and Cd^2+^ where the [BST pol] was ~12-fold greater than that of the primer-template [P/T]. The concentration of product DNA obtained at different [dTTP] were plotted against reaction time and fitted to Eq 1 to yield *k*_obs_ at each [dTTP]. The *k*_obs_ values were then plotted against [dTTP] which yielded a *k*_pol_ of 20 ± 2 s^−1^ and *K*_d,app_ of 26 ± 11 μM with Mg^2+^. The *k*_pol_ values obtained using Co^2+^, Mn^2+^ and Cd^+2^ were 123 ± 12, 166 ± 14, and 15 ± 1 s^−1^ respectively. Surprisingly, *K*_d,app_ values for the correct nucleotide incorporation were very similar with all metal ions varying between 15–28 μM. Figures 2 and 3 show the plots for dTMP incorporation opposite dA in the presence of 10 mM Mn^2+^, Mg^2+^, Co^2+^, and Cd^2+^ respectively. The results, summarized in Table 2, show that incorporation efficiencies for dTMP opposite dA (DNA_13A_) with Co^2+^, Mn^2+^, and Cd^2+^ were increased by ~6-, ~10-, and 1.3-fold respectively compared to Mg^2+^.

### Single-turnover kinetics for incorrect nucleotide incorporation by BST pol

**Figure 4:**
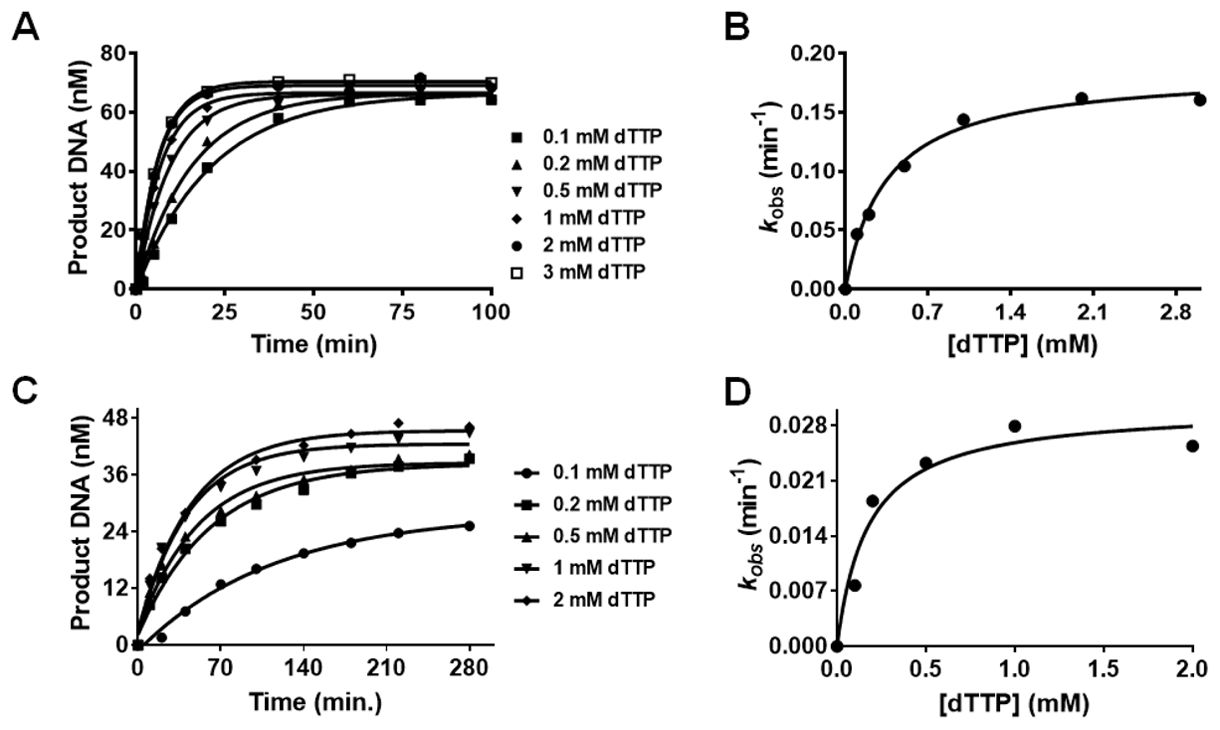
Concentration dependence of the rate of dTTP incorporation opposite dC as the templating base with Co^2+^, and Cd^2+^. BST pol (1 μM) was pre-incubated with DNA_13C_ (80 nM) in reaction buffer and was mixed with increasing concentrations of dTTP [100, 200, 500, 1000, 2000, and 3000 μM] containing 10 mM Co^2+^ or dTTP [100, 200, 500, 1000, and 2000 μM] containing 10 mM Cd^2+^. Reactions were quenched with 0.5 M EDTA (pH 8.0) at various times ranging from 1–100 min. with Co^2+^, and 10–280 min. with Cd^2+^. All data were obtained at 23 °C. (A), (C) plots of the amount of extended DNA product obtained as a function of time at various [dTTP] with 10 mM Co^2+^, or 10 mM Cd^2+^ respectively. Points are experimental, while curves are based on a fit of the data to Eq 1. (B), (D) the single exponential rates obtained were plotted as a function of [dTTP] and fitted to Eq 2 to obtain a *k*_pol_ of 0.18 ± 0.01 s^−1^ and a *K*_d,app_ of 354 ± 59 μM with 10 mM Co^2+^, a *k*_pol_ of 0.03 ± 0.002 s^−1^ and a *K*_d,app_ of 174 ± 60 μM with 10 mM Cd^2+^ respectively.

Single-turnover experiments were carried out with incorrect incoming dNTPs to study the effect of Co^2+^, Mn^2+^, and Cd^2+^ on base selectivity. Purine:pyrimidine, and pyrimidine:pyrimidine mismatches were tested. Table 2 summarizes the kinetic parameters obtained with these mispairs in the presence of Mg^2+^, Co^2+^, Mn^2+^, and Cd^2+^. The *k*_pol_ values obtained using Mg^2+^, Co^2+^, Mn^2+^ and Cd^2+^ for dAMP incorporation opposite dC were 0.07 ± 0.01, 0.30 ± 0.02, 9 ± 1, and 0.080 ± 0.004 s^−1^ respectively. The *K*_d,app_ value with Mg^2+^ was relatively high (3000 μM) compared to other divalent cations whereby the *K*_d,app_ values varied between 67–293 μM. In general, for dAMP incorporation opposite dC, Mg^2+^ showed the greatest base selectivity with all the mispairs tested followed by Co^2+^, Cd^2+^, and Mn^2+^ (Table 2). The reduction in base selectivity with Mn^2+^ was a direct result of a dramatic increase in *k*_pol_ with the incorrect incoming dATP. Similar trends were observed when dATP was replaced by dTTP with all divalent cations that were tested (Table 2). Figure 4 shows the plots for dTMP incorporation opposite dC in the presence of Co^2+^, and Cd^2+^ respectively.

### Effect of metal ions on ground-state binding of dNTPs to pol/P/T binary complexes with Mg^2+^, Mn^2+^, Co^2+^, and Cd^2+^

**Figure 5:**
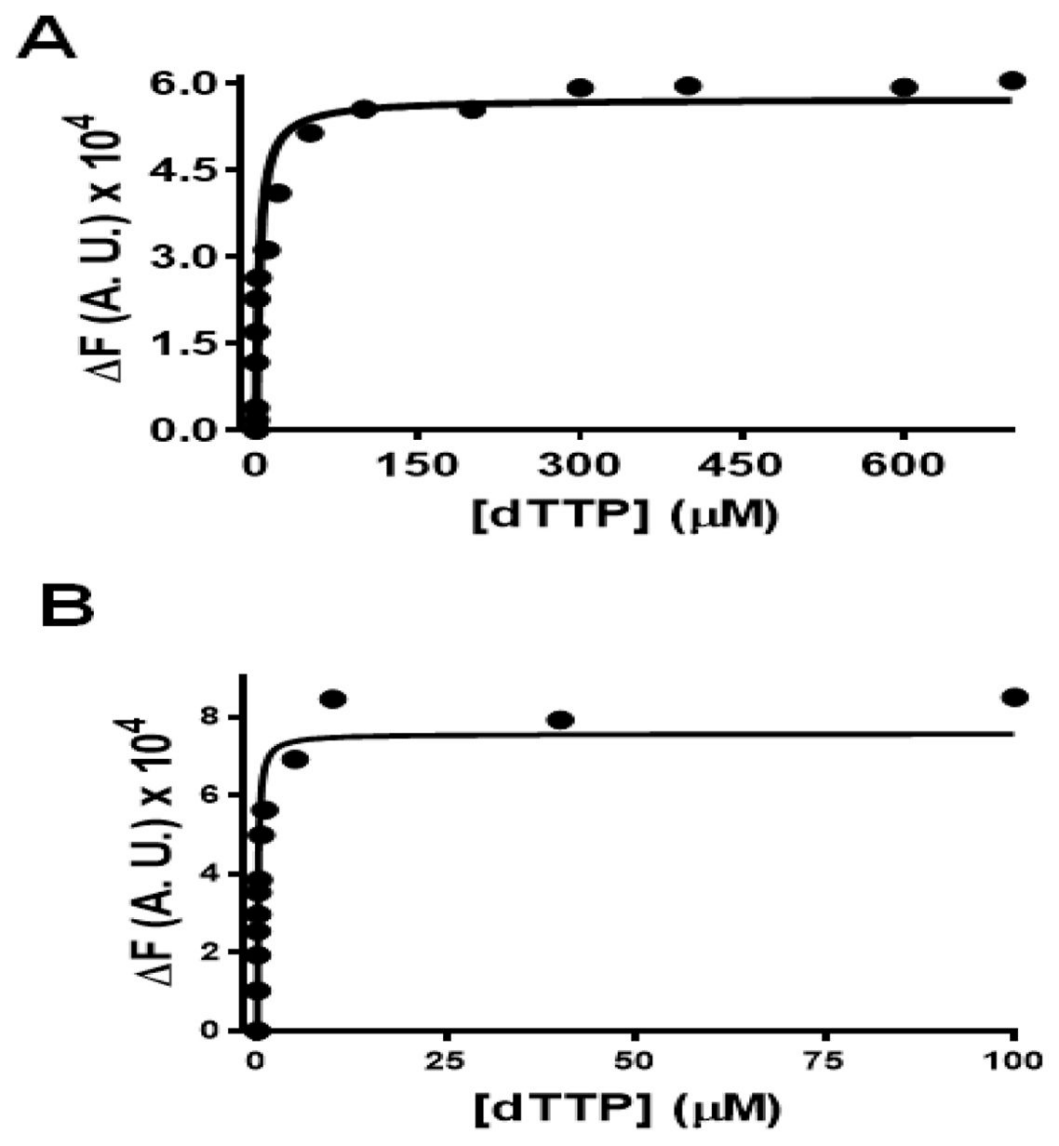
Equilibrium fluorescence titration of the BSTpol-ddP/T complex with increasing [dTTP] in the presence of Co^2+^ and Cd^2+^. The concentration of DNA_Pdd_ was 200 nM and that of BST pol was 1 μM. (A) plot showing the change in fluorescence quenching as a function of [dTTP] in the presence of Co^2+^. The concentrations of dTTP used were 0, 0.005, 0.01, 0.02, 0.1, 0.5, 2, 10, 20, 50, 100, 200, 300, 400, 500, 600 and 700 μM. Fluorescence intensities at 365 nm were fitted to a hyperbolic equation. Titration of dTTP vs. 2AP in the presence of 10 mM Co^2+^ gives a *K*_d,g_ = 3 ± 1 μM. (B) plot showing the change in fluorescence quenching as a function of [dTTP] in the presence of Cd^2+^. The concentrations of dTTP used were 0, 0.005, 0.01, 0.02, 0.05, 0.1, 0.25, 0.5, 1, 5, 10, 40 and 100 μM. Fluorescence intensities at 365 nm were fitted to a hyperbolic equation to obtain a *K*_d,g_ = 0.4 ± 0.1 μM. (Δ*F*) represents the change in fluorescence in the direction of quenching and Δ*F* increases with an increase in [dTTP].

**Scheme 1:**
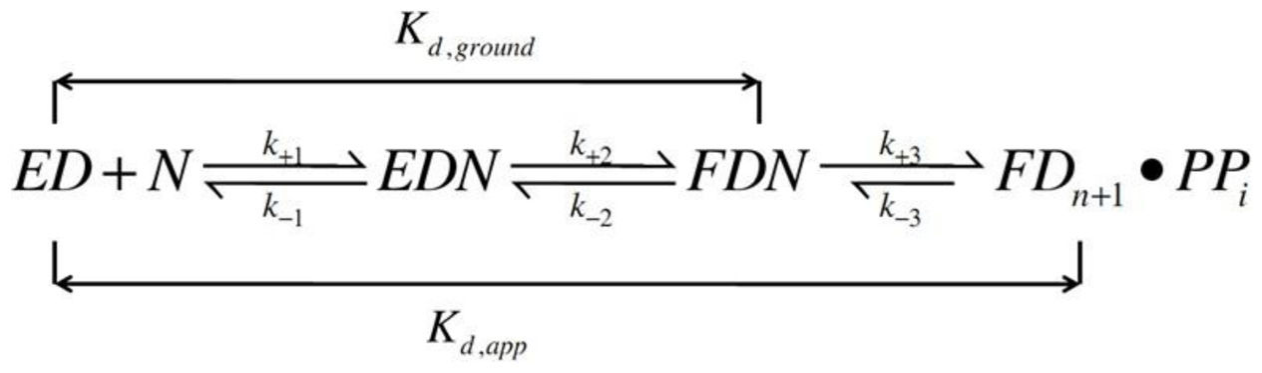
Minimal kinetic scheme depicting the ground state binding affinity (*K*_d,g_) and apparent binding affinity (*K*_d,app_) for an incoming dNTP.

We then investigated the effect of Mg^2+^, Co^2+^, Mn^2+^, and Cd^2+^ on ground-state binding affinity (*K*_d,g_) of the incoming dNTPs. Equilibrium fluorescence titrations were carried out using a P/T containing 2AP as the templating base (DNA_Pdd_) and the *K*_d,g_ values for dTTP binding (opposite 2AP) to a ddP/T-BSTpol binary complex were measured. The *K*_d,g_ value for dTTP binding opposite 2AP was determined by measuring the change in 2AP fluorescence observed as a function of [dTTP] in the presence of different metal ions. Scheme 1 shows the minimal kinetic scheme depicting the ground state binding affinity (*K*_d,g_) and apparent binding affinity (*K*_d,app_) for an incoming dNTP. In the presence of Mg^2+^, the *K*_d,g_ value for dTTP binding opposite 2AP was determined to be 1 μM (Table 3). *K*_d,g_ values obtained in the presence of Co^2+^, Mn^2+^, and Cd^2+^ were 3.3 ± 0.3, 0.10 ± 0.02, and 0.4 ± 0.1 μM respectively. Figure 5, panels A and B, show equilibrium fluorescence titration results for the BSTpol-ddP/T complex with increasing [dTTP] in the presence of Co^2+^ and Cd^2+^.

**Figure 6:**
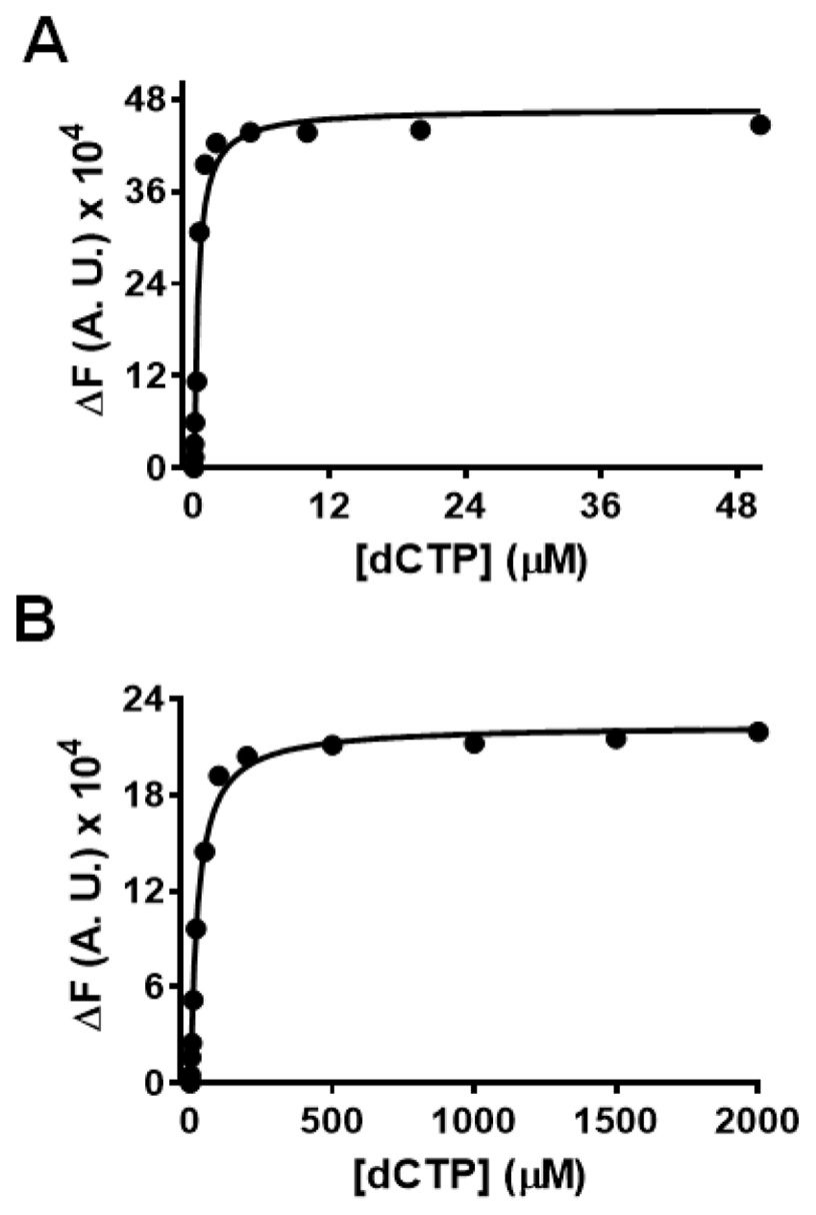
Equilibrium fluorescence titration of the BST-ddP/T complex with increasing [dCTP] in the presence of Mn^2+^, and Co^2+^. The concentration of DNA_Pdd_ was 200 nM and that of BST pol was 1 μM. (A) plot showing the change in fluorescence quenching as a function of [dCTP] in the presence of Mn^2+^. The concentrations of dCTP used were 0, 0.01. 0.02, 0.04, 0.1, 0.25, 0.5, 1, 2, 5, 10, 20 and 50 μM. Fluorescence intensities at 365 nm were fitted to a hyperbolic equation to obtain a *K*_d,g_ = 0.3 ± 0.1 μM. (B) plot showing the change in fluorescence quenching as a function of [dCTP] in the presence of Co^2+^. The concentrations of dCTP used were 0, 0.5, 1, 2, 5, 10, 20, 50, 100, 200, 500, 1000, 1500, and 2000 μM. Fluorescence intensities at 365 nm were fitted to a hyperbolic equation to obtain a *K*_d,g_ = 25 ± 2 μM. (Δ*F*) represents the change in fluorescence in the direction of quenching and Δ*F* increases with an increase in [dCTP].

We next tested whether BST pol is able to similarly discriminate against an incorrect incoming dNTP in the presence of these metal ions. The change in 2AP fluorescence was measured as a function of [dCTP] to determine the *K*_d,g_ value for dCTP binding opposite 2AP. The *K*_d,g_ values obtained for dCTP binding in the presence of Mg^2+^, Co^2+^, and Mn^2+^ were 1463 ± 130 μM, 25 ± 2 μM, and 0.34 ± 0.08 μM respectively. These data clearly demonstrate that compared to Mg^2+^, Co^2+^ and Mn^2+^ provide a ~60-fold and ~5000-fold tighter ground-state binding of an incorrect dNTP substrate (dCTP) respectively. Figure 6, panels A and B, show equilibrium fluorescence titrations for the BST pol-ddP/T complex with increasing [dCTP] in the presence of Mn^2+^ and Co^2+^.

### Extension beyond a mismatched P/T with Mg^2+^, Mn^2+^, Co^2+^ and Cd^2+^

We next determined how efficiently BST pol could extend a duplex DNA containing a mismatch at the P/T terminus in the presence of Mg^2+^, Mn^2+^, Co^2+^ and Cd^2+^. We chose a DNA oligo containing a dA/dC mismatch (DNA_ACMM_) as this represents a purine-pyrimidine pair containing a nascent wobble mispair having the correct size but with distorted geometry. As compared to extension past a matched base-pair at the primer terminus, where the *k*_pol_ was 20 ± 2 s^−1^ and the *K*_d,app_ was 26 ± 11 μM, the extension past a duplex DNA containing a mismatch at the P/T terminus in the presence of Mg^2+^ resulted in a *k*_pol_ of 0.13 ± 0.01 s^−1^ and a *K*_d,app_ value of 618 ± 185 μM. This sharp decrease in the *k*_pol_ value (200-fold) accompanied by a substantial increase in the *K*_d,app_ value (24-fold) results in ~4800-fold decrease in the incorporation efficiency of dNMP incorporation when BST pol encounters a dA/dC mismatch at the primer terminus in the presence of Mg^2+^.

With Co^2+^, the *K*_d,app_ was 1100 μM, higher than that obtained in the presence of Mg^2+^, but the *k*_pol_ was 10-fold higher, showing that the incorporation of correct dNMP past the mismatched DNA was more readily accomodated compared to Mg^2+^. In the presence of Mn^2+^, the incorporation efficiency of correct dNMPs past the dA/dC mismatch was 100-fold higher than with Mg^2+^, demonstrating that the correct dNMP was much more readily incorporated past an dA/dC mismatch compared to Mg^2+^. This enhanced incorporation efficiency with Mn^2+^ directly resulted from a 20-fold increase in the *k*_pol_ and a ~5-fold decrease in the *K*_d,app_ values. When Mg^2+^ was replaced with Cd^2+^ in the assay, saturation with the correct incoming dNTP was not achieved so we were unable to determine the actual *k*_pol_ and *K*_d,app_ values. Consequently we had to plot the rates observed versus [dNTP] and determined the *k*_pol_/*K*_d,app_ value from the slope (Eq 3). Compared to Mg^2+^, the incorporation efficiency of the correct dNTP showed a marginal increase (1.4-fold) in the presence of Cd^2+^ suggesting that, unlike Co^2+^ and Mn^2+^, Cd^2+^ does not promote extension past a dA/dC mismatch. The kinetic parameters for extension past a dA/dC mismatch are summarized in Table 4.

### Mg^2+^, Co^2+^, Mn^2+^, and Cd^2+^ can support processive DNA synthesis with BST pol

**Figure 7:**
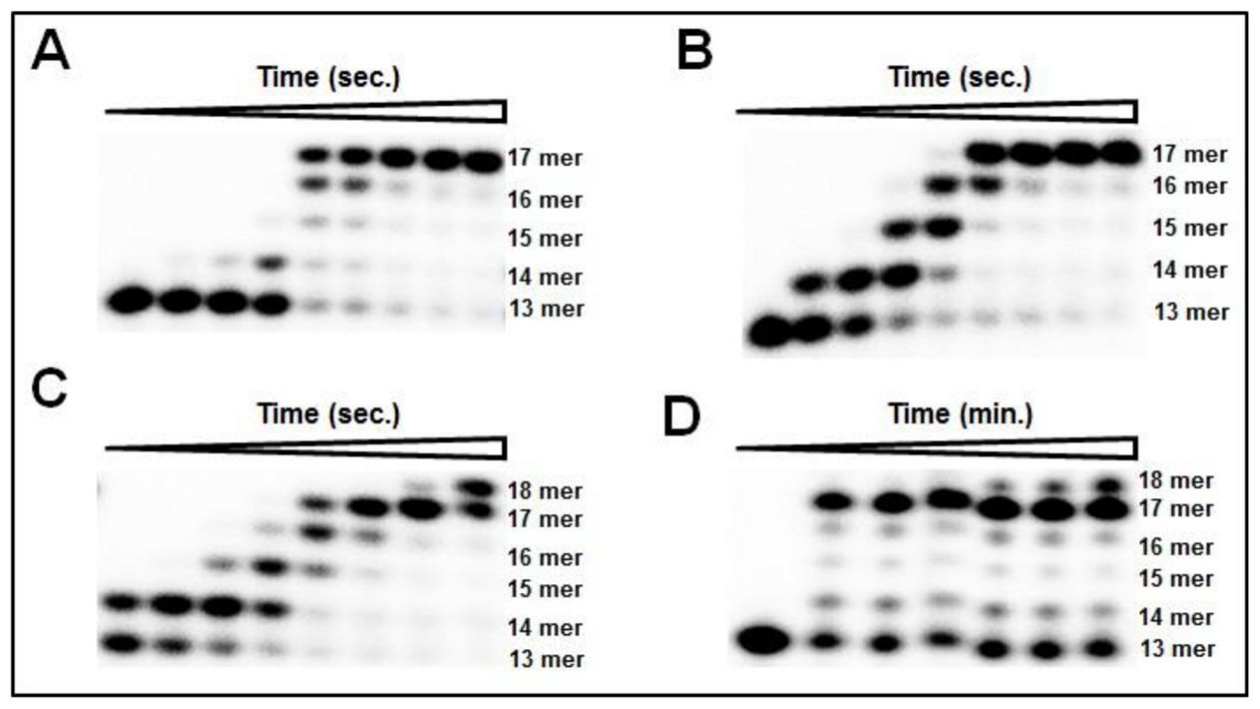
Processive DNA synthesis with Mg^2+^, Mn^2+^, Co^2+^, and Cd^2+^. BST pol (1 μM) was incubated with 80 nM DNA_13A_ and 200 μM dNTP mix in buffer containing 10 mM Mg^2+^, Co^2+^, or Cd^2+^ and aliquots were taken out at the noted times. (A) phosphorimages of the products of DNA_13A_ full length extension using BST pol in the presence of 10 mM Mg^2+^ at various times: 0, 0.005, 0.01, 0.05, 0.1, 0.5, 1, 5, and 30 s. (B) phosphorimages of the products of DNA_13A_ full length extension using BST pol in the presence of 10 mM Co^2+^ at various times: 0, 0.005, 0.01, 0.05, 0.1, 0.5, 1, 5, and 30 s. (C) phosphorimages of the products of DNA_13A_ full length extension using BST pol in the presence of 10 mM Mn^2+^ at various times: 0.005, 0.01, 0.05, 0.1, 0.5, 1, 5, and 30 s. (D) phosphorimages of the products of DNA_13A_ full length extension using BST pol in the presence of 10 mM Cd^2+^ at various times: 0, 0.5, 2, 5, 10, 15, and 30 min. Full extension assays with Mg^2+^, Mn^2+^, and Co^2+^ were carried out using RQF while assays with Cd^2+^ were carried out on the benchtop.

We tested whether these divalent cations could support processive DNA synthesis by incubating BST pol with DNA_13A_ and all four dNTPs, quenching the reaction at various times. Figure 7 panels A–D shows the full extension products obtained with Mg^2+^, Mn^2+^, Co^2+^, and Cd^2+^. The results show that all four metal ions were able to support processive DNA synthesis with BST pol. As expected with Mg^2+^, a fully duplexed DNA product was obtained. Intermediate products were observed from 0.05–1 s while majority of these intermediate length products were converted to full length product at the longer time points (5–30 s) (Figure 7, Panel A). Similar trends were observed when Mg^2+^ was replaced with Co^2+^ in the assay (Figure 7, Panel B). In contrast, when Mn^2+^ was used in the assay, up to 1 s only the full extension product was observed while at longer time points (5–30 s) an 18mer product was also found (Figure 7, Panel C). Similar to the results with Mn^2+^, the 18mer DNA product was also observed with Cd^2+^ (Figure 7, Panel D) indicating that in the presence of these two metal ions, template independent DNA synthesis can occur, in contrast to the results observed with Mg^2+^ and Co^2+^.

## 4. Conclusions

Several divalent cations including Co^2+^, Mn^2+^, and Ni^2+^ have been characterized as “mutagenic”, since they reduce the fidelity of DNA synthesis [3,9–13,15]. Previous attempts to address the reasons for the mutagenic behavior of these metal ions have mainly focused on Mn^2+^ [11,12]. For example, translesion DNA synthesis in herpes simplex virus Type I was promoted by Mn^2+^ [25]. Also, the rate of misincorporation opposite an abasic site by T4 DNA pol was enhanced by 11–34 fold when Mn^2+^ was substituted for Mg^2+^ [22]. In addition, studies on pol β using blunt-ended DNA showed primer-extension in the presence of Mn^2+^ rather than Mg^2+^ [4]. Moreover, the rates of incorporation of correct and incorrect nucleotides have not been determined under single-turnover conditions in the presence of different metal ions except for Mn^2+^ [22], and Co^2+^ [17].

We tested several divalent cations and found that, apart from Mg^2+^, only Mn^2+^, Co^2+^ and Cd^2+^ supported catalysis with BST pol. When Mg^2+^ was replaced by Co^2+^, the *k*_pol_ for the correct incoming dTTP vs. dA increased by 6-fold. Similarly, the *k*_pol_ value with Mn^2+^ was 8-fold higher and only slightly lower with Cd^2+^ compared to Mg^2+^. Despite the variation in *k*_pol_ values, surprisingly the *K*_d,app_ values were nearly identical with all four divalent cations. It is also worth noting that the efficiency of correct insertions (dTMP vs. dA) was greatest for Mn^2+^ followed by Co^2+^, Cd^2+^, and Mg^2+^ (Table 2). This trend observed with BST pol contrasts with the results obtained with RB69pol [14], where the highest incorporation efficiency was observed in the presence of Co^2+^ followed by Mn^2+^ and Mg^2+^. Nevertheless, the incorporation efficiency with both pols is much higher with Co^2+^ as compared to Mg^2+^. The possible reasons for this trend are discussed below.

With incorrect incoming dNTPs the *k*_pols_ were 1.5–14 times higher with Co^2+^ vs. Mg^2+^. The *K*_d,app_ values for the incorrect incoming dNTP were generally 7–11 fold lower with Co^2+^ vs. Mg^2+^. As a result, the incorporation efficiencies for incorrect incoming nucleotides were ~10–50 fold higher with Co^2+^ vs. Mg^2+^ suggesting that base discrimination is greatly reduced with Co^2+^ vs. Mg^2+^. This trend observed with BST pol stands in contrast to similar studies carried out using RB69pol [14] whereby the *K*_d,app_ values did not show any clear trend (e.g. the *K*_d,app_ value for dATP binding opposite dC was nearly identical with both Mg^2+^ and Co^2+^, slightly lower with Co^2+^ with dTTP vs. dC and much higher with Co^2+^ when dGMP incorporation was measured opposite dA). A similar trend was observed with Cd^2+^. This decrease is much less pronounced compared to the situation where Mn^2+^ was used in place of Mg^2+^ whereby the *k*_pols_/*K*_d,app_ values were 13–1300 fold higher as compared to Mg^2+^ with the largest differences observed when dAMP was incorporated vs. dC (*k*_pol_ = 9 s^−1^ and *K*_d,app_ = 293 μM with Mn^2+^ compared to *k*_pol_ = 0.07 s^−1^ and *K*_d,app_ = 3000 μM with Mg^2+^). Interestingly, most of the enhancement in incorporation efficiencies resulted from a sharp increase in *k*_pol_ values in the presence of Mn^2+^. Surprisingly, the *k*_pol_ values for misincorporation were similar for both Cd^2+^ and Mg^2+^. Moreover, the *K*_d,app_ values were 4–50 fold lower with Cd^2+^ vs. Mg^2+^ suggesting that Cd^2+^ reduces base selectivity.

Wang et al. [26] have provided structural evidence for the rare tautomer hypothesis of spontaneous mutagenesis using the D598A/F710Y double mutant of BST pol. They showed that in the presence of Mg^2+^ when dCMP incorporation was observed opposite dT, the polymerase adopts an “ajar” or partially closed conformation which prevents the mismatch incorporation. Additionally, the triphosphate tail of dCTP is not properly aligned for catalysis. In contrast, in the presence of Mn^2+^, the C/A mismatch adopts a tautomeric cognate base-pair shape, that is virtually indistinguishable from a canonical, Watson-crick base pair in double stranded DNA in the insertion site [26]. Moreover, the triphosphate tail is properly aligned for catalysis, and the polymerase is also observed in the closed conformation. These results support our kinetic data when we had dATP opposite dC with Mg^2+^ and Mn^2+^ where the *k*_pol_ value was ~130-fold higher with Mn^2+^ vs. Mg^2+^. This could explain why the rates of misincorporation are higher in the presence of Mn^2+^ compared to Mg^2+^. Similarly, the triphosphate tail is likely to be in the proper orientation for catalysis in the presence of Co^2+^ and mimics the T/A Watson-crick base pair explaining the higher *k*_pol_ values observed with Co^2+^ vs. Mg^2+^. Even though crystal structures with bound Co^2+^ and Cd^2+^ are currently not available, based on the kinetic parameters we can speculate that, similar to Mn^2+^, the C/A mismatch adopts a tautomeric cognate base-pair shape and the triphosphate tail is likely to be properly aligned for catalysis with Co^2+^ and Cd^2+^. This could explain the higher *k*_pol_ values and lower *K*_d,app_ values observed with these divalent cations. Support for this notion will require further crystal structure evidence which could directly show that a C/A mismatch adopts a cognate shape mimic for BST pol in the presence of Co^2+^ and Cd^2+^.

With respect to the ground-state binding affinities for dTTP vs. dAP, as measured by 2AP fluorescence quenching, the lowest dissociation constant (*K*_dg_) was observed with Mn^2+^/BSTpol complex followed by Cd^2+^, Mg^2+^ and then Co^2+^. Surprisingly, with BST pol, in the presence of Co^2+^, the *K*_d,g_ value for dTTP binding opposite 2AP was 3-fold higher as compared to Mg^2+^ but with RB69pol, the *K*_d,g_ value was 5-fold lower [17]. For an incorrect dNTP (dCTP vs. 2AP) there was a difference of 1400-fold in *K*_d,g_ values with Mg^2+^ as compared to dTTP vs. 2AP which clearly shows that, in the presence of Mg^2+^, BST pol discriminates well between the correct and incorrect incoming dNTP. This trend is very similar to that observed with RB69pol even though the discrimination is more selective with BST pol (1400-fold) compared to RB69pol (40-fold) suggesting that these pols behave very differently in terms of their ability to discriminate among the incoming dNTPs. In contrast, discrimination in ground state binding affinity sharply dropped to only 8-fold and 3-fold with Co^2+^ and Mn^2+^ respectively. In the presence of Co^2+^, the *K*_d,g_ for dCTP binding opposite 2AP was much lower (25 μM) with BST pol as compared to RB69pol where the *K*_d,g_ value could not be determined as saturation with dCTP was not attained. With Mn^2+^, this value was ~5000-fold tighter as compared to Mg^2+^ and ~80-fold tighter compared to Co^2+^. Clearly, the difference in ground-state binding affinities observed with BST pol with Mg^2+^, Mn^2+^, Co^2+^, and Cd^2+^ are more varied compared to similar data obtained with RB69pol [14].

### Primer extension past dA/dC mismatch

The number of errors in nascent DNA can be increased if the DNA pol extends beyond a mismatch at a P/T terminus [27]. When we investigated the influence of divalent cations on the ability of BST pol to extend the primer past a dA/dC mismatch, we found that the efficiency of incorporation was highest with Mn^2+^ followed by Co^2+^, Cd^2+^, and Mg^2+^ (Table 4). This trend mainly resulted from an increase in *k*_pol_ values with Mn^2+^, and Co^2+^ compared to Mg^2+^ while the *K*_d,app_ values did not show a great deal of variation. On the other hand, when a dA/dC mismatch was extended by BST pol with Cd^2+^, the incorporation efficiency was marginally higher as compared to Mg^2+^. Since saturation was not achieved with incoming dNTP we only determined the *k*_pol_/*K*_d_ value with Cd^2+^. The structural evidence for rare tautomer hypothesis provided by Wang et al. [26] also supports the mismatch bypass experimental data. The 20-fold higher *k*_pol_ value obtained in the presence of Mn^2+^ could be explained on the basis of the formation of dA/dC tautomeric cognate base-pair at the P/T junction, which resembles a canonical dA/dT base pair and hence the polymer is likely not able to sense the presence of a mismatch at the primer terminus and is able to readily extend past the mismatch. On the other hand, in the presence of Mg^2+^, the dA/dC mismatch forms a wobble base pair at the primer terminus and the polymerase is not able to readily incorporate the incoming dNTP past this mismatch resulting in reduced *k*_pol_ values. This explanation could also be extended to the kinetic data obtained with Co^2+^ resulting in a 10-fold increase in the *k*_pol_ value as compared to the situation when Mg^2+^ is the divalent cation.

### Effect of active metal ions on full extension with BST pol

We tested the ability of four divalent cations to support processive DNA synthesis and found that BST pol is able to carry out full length primer extension with Mg^2+^, Mn^2+^, Co^2+^, and Cd^2+^. In particular, the results obtained with Mn^2+^ and Cd^2+^ are quite interesting as we observed 18 mer products with both these metal ions even though a 13/17 mer DNA P/T was utilized suggesting that BST pol is able to carry out template independent DNA synthesis in the presence of these two metal ions. Similar results have been reported by Pelletier et al. with pol β [4] where they observed primer extension in crystal soaking experiments in the presence of Mn^2+^ and Cd^2+^.

### Comparison of the properties of various divalent cations

Several properties of divalent cations such as their ability to lower the p*K*_a_ of the 3′ OH group, their ionic radii, and their coordination geometry preferences are crucial in order to determine if a given divalent cation could serve as a cofactor for DNA pols. One of the most important factors appears to be the ability of the divalent cation to enhance the nucleophilicity of the 3′ OH group of the primer by lowering its p*K*_a_ so that it can attack the α-phosphate atom of the incoming dNTP. In this respect, the effect on reducing the p*K*a of bound water is lowest for Fe^2+^ (8.4), and is about the same for Co^2+^, Cd^2+^, and Zn^2+^ (9.4, 9.8, and 9.6 respectively), slightly higher for Mn^2+^, and Ni^2+^ (10.1 and 10.6), and considerably higher for Mg^2+^ and Ca^2+^ (11.4 and 12.8) (Table 5). Based on these values, Co^2+^, Cd^2+^, and Zn^2+^ would be expected to be more effective as cofactors but that is clearly not the case as Zn^2+^ is not active at all with BST pol. Moreover, Ni^2+^ is also not able to support the polymerase activity of BST pol despite having a comparable p*K*_a_ value. On the other hand, Ca^2+^ does not lower the p*K*_a_ of the 3′ OH group of the primer and thus is not able to support catalysis except for Dpo4 which is the only pol able to utilize Ca^2+^ [6]. Surprisingly, Fe^2+^ provides the maximum decrease in the p*K*_a_ of the 3′ OH group but is ineffective as a cofactor for BST pol or RB69pol [17]. Hence, the ability of divalent cations to lower the p*K*_a_ of the 3′ OH group is not the sole factor for promoting catalysis. For example, the divalent cation occupying the “A site” helps to determine the proximal distance between the α-phosphorous atom of the incoming dNTP and the 3′-hydroxyl group during the transition state. The ionic radii of Mg^2+^ is 0.86 Å, very similar to that of Mn^2+^, Co^2+^, Ni^2+^ and Zn^2+^, while the ionic radii of Cd^2+^ and Fe^2+^ is slightly higher (0.95 and 0.92 Å). Based on similar values, the ionic radii of Mg^2+^, Mn^2+^, Co^2+^, Ni^2+^, and Zn^2+^ should allow these metal ions to bring the 3′-hydroxyl group and α-phosphate atom of the incoming dNTP close enough for reaction but despite this, Ni^2+^ and Zn^2+^ fail to support catalysis with BST pol and Ni^2+^ is only a weak cofactor for RB69pol [17]. In contrast, Cd^2+^ does support BST pol activity and the *k*_pol_ value with Cd^2+^ is only slightly lower than the value obtained with Mg^2+^ while Fe^2+^ whose ionic radii is very similar to Cd^2+^ fails to catalyze nucleotidyl transfer reaction. The ionic radii of Ca^2+^ is 1.1 Å, thus accounting for its inability to bring the 3′-hydroxyl group and α-phosphate atom of the incoming dNTP within range for the reaction to occur so Ca^2+^ is not able to support catalysis with almost all pols except for Dpo4 [17].

Apart from these factors, the preference of different divalent cations for a given coordination geometry plays a critical role in the nucleotidyl transfer reaction. Studies by Xia et al. [7] showed that the metal ions (Mg^2+^ and Mn^2+^) present in the B site is in perfect octahedral geometry while the metal ion in the A site is present in a distorted octahedral geometry. In addition to Mg^2+^ and Mn^2+^, Co^2+^, Cd^2+^, Fe^2+^, and Ni^2+^ are also able to form octahedral complexes and can potentially support the reaction with BST pol but Fe^2+^, and Ni^2+^ are not cofactors for BST pol. Ca^2+^ can also form octahedral complexes but due to its much larger ionic radii it prefers pentagonal bipyramidal and hexagonal bipyramidal geometries. The inability of Zn^2+^ to act as a cofactor for BST pol despite its ability to effectively lower the p*K*a of hydroxyl group and its similar ionic radii compared to Mg^2+^ could be explained on the basis of its preference for tetrahedral as opposed to octahedral geometry. The ability of only selected divalent cations (including Mg^2+^, Co^2+^, Mn^2+^, and Cd^2+^) to act as cofactors for BST pol is consistent with: 1) their ability to effectively lower the p*K*a of the water molecule (and presumably the 3′-hydroxyl group of the primer); 2) similar ionic radii of these metal ions; 3) the ability of these metal ions to form octahedral complexes [28]. The highly mutagenic behavior of Mn^2+^ as compared to Mg^2+^ can be explained on the basis of the more polarizable nature of Mn^2+^ [28]. Also, in the context of a hexahydrated metal complex (Mn[H_2_O]_6_^2+^, Mg[H_2_O]_6_^2+^) there is a greater energy penalty with Mg^2+^ compared to Mn^2+^, when the inner sphere coordination number is changed from 6→5→4, indicating more rigid coordination requirements for Mg^2+^ complexes compared to Mn^2+^. This in turn allows dNTPs (correct and incorrect) to be accessible to the nucleotide binding pocket with Mn^2+^ [29]. In addition, the ability of transition metal ions to bind more tightly to the triphosphate moiety of dNTPs and carboxylate groups as compared to Mg^2+^, could explain the lower base selectivity in the presence of Mn^2+^ [4].

### Comparison of metal ion preferences for different DNA polymerases

Mg^2+^ is the universal metal ion cofactor for all DNA polymerases. Even though Mg^2+^ is preferred by pols, Mn^2+^ and Co^2+^ can also be used in place of Mg^2+^ but this usually results in a decrease in replication fidelity [9–11,15]. Crystal soaking experimental studies on pol β have shown that Cd^2+^, and Zn^2+^ were able to act as cofactors of pol β [4] while one study shows that BST pol was only able to utilize Cd^2+^. On the other hand, both these divalent cations were inactive with RB69pol [14]. Ni^2+^ has been shown to support catalysis for all DNA polymerases albeit with greatly reduced activity except for human pol α [30], human pol β [4], and Dpo4 [6]. Our results show that Ni^2+^ is also not able to activate BST pol. Interestingly, Fe^2+^ exhibits properties conducive to supporting catalysis with pols but fails to catalyze nucleotidyl transfer with BST pol, RB69pol [17,31] and HSV-1 pol [32,33] but the reason for this is not clear.

In summary, Mg^2+^ is the best divalent cation cofactor for pols in terms of the rate of nucleotidyl transfer reaction, base selectivity, exonuclease activity, and the efficiency of extension past mismatched DNA. Other divalent cations can substitute for Mg^2+^ but they affect the various fidelity checkpoints in the minimal kinetic scheme (Scheme 1) and their effect varies with the nature of the metal ion. Our results, together with previous studies on other pols, show that DNA polymerases from different families show preferences for different metal ions but we cannot predict, as yet, which ones will work based solely on their properties that we have described above.

## Figures and Tables

**Table 1:** Metal ion preferences for activation of BST DNA polymerase.

Active	Inactive
Mg^2+^, Co^2+^, Mn^2+^, Cd^2+^	Fe^2+^, Ca^2+^, Zn^2+^, Ni^2+^, Sr^2+^, Ba^2+^, Cu^2+^, Cr^2+^

**Table 2:** Summary of pre-steady-state kinetic parameters for incorporation of dNMPs by BST pol using different metal ions with 66 mM Tris-HCl (pH 7.3) at 23 °C.

Metal ion	dNTP	Template	*k*_pol_ (s^−1^)	*K*_d,app_ (mM)	*k*_pol_/*K*_d,app_ (mM^−1^ s^−1^)
Mg^2+^	dTTP	dA	20	26	7.7 × 10^−1^
	dATP	dC	0.07	3000	2.3 × 10^−5^
	dTTP	dC	0.002	2600	7.5 × 10^−7^
Co^2+^	dTTP	dA	123	28	4.4
	dATP	dC	0.3	267	1.1 × 10^−3^
	dTTP	dC	0.003	354	8.5 × 10^−6^
Mn^2+^	dTTP	dA	166	22	7.5
	dATP	dC	9	293	3.1 × 10^−2^
	dTTP	dC	0.004	396	1.0 × 10^−5^
Cd^2+^	dTTP	dA	15	15	1
	dATP	dC	0.08	67	1.2 × 10^−3^
	dTTP	dC	0.0005	174	2.9 × 10^−6^

Standard deviations were within 10–20% and ~30% for *k*_pol_ and *K*_d,app_ values, respectively.

**Table 3:** Equilibrium fluorescence titration data for BST pol using Mg^2+^, Co^2+^, Mn^2+^, and Cd^2+^ with 2AP as the templating base.

Metal ion	P/T	dNTP	*K*_d,g_ (μM)
Mg^2+^	(ddP/T)	dTTP	1
Mg^2+^	(ddP/T)	dCTP	1463
Co^2+^	(ddP/T)	dTTP	3
Co^2+^	(ddP/T)	dCTP	25
Mn^2+^	(ddP/T)	dTTP	0.1
Mn^2+^	(ddP/T)	dCTP	0.3
Cd^2+^	(ddP/T)	dTTP	0.4
Cd^2+^	(ddP/T)	dCTP	ND

The concentrations of Mg^2+^, Co^2+^, Mn^2+^, and Cd^2+^ used in the titrations were 10 mM each. Additional details are provided in the text. ND = not determined.

**Table 4:** Pre-steady-state kinetic parameters for extension beyond dA/dC mismatch for BST pol using Mg^2+^, Mn^2+^, Co^2+^ and Cd^2+^ where dAMP is being inserted opposite a templating dT.

Metal ion	*k*_pol_ (s^−1^)	*K*_d,app_ (μM)	*k*_pol_/*K*_d,app_ (μM^−1^ s^−1^)
Mg^2+^	0.1	618	1.6 × 10^−4^
Co^2+^	1	1100	9.1 × 10^−4^
Mn^2+^	2	127	1.6 × 10^−2^
Cd^2+^			2.3 × 10^−4^^a^

a Represents the ratio of the *k*_pol_ to the *K*_d,app_ value as saturation is not achieved with the incoming dNTP.

Standard deviations were within 10–20% and ~30% for *k*_pol_ and *K*_d,app_ values, respectively.

**Table 5:** Ionic radii, coordination geometry, and p*K*a of water molecules coordinated to (Mg^2+^, Co^2+^, Mn^2+^, Cd^2+^, Fe^2+^, Ni^2+^, Zn^2+^, and Ca^2+^).

Metal ion								
	Mg^2+^	Co^2+^	Mn^2+^	Cd^2+^	Fe^2+^	Ni^2+^	Zn^2+^	Ca^2+^
Ionic radius (Å)	0.86	0.89	0.81	0.95	0.92	0.83	0.88	1.1
Coordination geometry^a^	Oct	Oct	Oct	Oct	Oct	Oct	Oct	Oct
	Td	Td	Td	Td	Td	Td	Td^a^	Td
	Sq	Sq		HBP		TBP	TBP	HBP
	TBP	TBP						
p*K*_a_ of the water molecule	11.4	9.4	10.1	9.8	8.4	10.6	9.6	12.8

Td represents Tetrahedral; Sq represents Square planar; TBP represents Trigonal bipyramidal; Oct represents Octahedral; PBP represents pentagonal bipyramid; and HBP represents hexagonal bipyramidal.

a Even though Zn^2+^ can form octahedral complexes, the majority of Zn^2+^ complexes are tetrahedral.

## Abbreviations

2AP: 2-aminopurine
BST pol: bacillus stearothermophilus DNA polymerase
dNTP: deoxynucleoside triphosphate
Dpo4: DNA polymerase IV from *Sulfolobus solfataricus*
k_obs_: observed rate constant
*k*_pol_: maximum rate of dNMP incorporation
*K_d,app_*: apparent equilibrium dissociation constant for [dNTP] that supports the half-maximal rate of dNMP incorporation
*K*_dg_: ground-state equilibrium dissociation constant for an incoming dNTP from a DNA pol
DNA: dNTP ternary complex
pol: DNA polymerase
